# Systemic, Local, and Imaging Biomarkers of Brain Injury: More Needed, and Better Use of Those Already Established?

**DOI:** 10.3389/fneur.2015.00026

**Published:** 2015-02-18

**Authors:** Keri L. H. Carpenter, Marek Czosnyka, Ibrahim Jalloh, Virginia F. J. Newcombe, Adel Helmy, Richard J. Shannon, Karol P. Budohoski, Angelos G. Kolias, Peter J. Kirkpatrick, Thomas Adrian Carpenter, David K. Menon, Peter J. Hutchinson

**Affiliations:** ^1^Division of Neurosurgery, Department of Clinical Neurosciences, University of Cambridge, Cambridge, UK; ^2^Wolfson Brain Imaging Centre, Department of Clinical Neurosciences, University of Cambridge, Cambridge, UK; ^3^Division of Anaesthesia, Department of Medicine, University of Cambridge, Cambridge, UK

**Keywords:** acute brain injury (human), biomarkers, cerebral energy metabolism, cell death, inflammation, blood–brain barrier, multimodality monitoring, imaging

## Abstract

Much progress has been made over the past two decades in the treatment of severe acute brain injury, including traumatic brain injury and subarachnoid hemorrhage, resulting in a higher proportion of patients surviving with better outcomes. This has arisen from a combination of factors. These include improvements in procedures at the scene (pre-hospital) and in the hospital emergency department, advances in neuromonitoring in the intensive care unit, both continuously at the bedside and intermittently in scans, evolution and refinement of protocol-driven therapy for better management of patients, and advances in surgical procedures and rehabilitation. Nevertheless, many patients still experience varying degrees of long-term disabilities post-injury with consequent demands on carers and resources, and there is room for improvement. Biomarkers are a key aspect of neuromonitoring. A broad definition of a biomarker is any observable feature that can be used to inform on the state of the patient, e.g., a molecular species, a feature on a scan, or a monitoring characteristic, e.g., cerebrovascular pressure reactivity index. Biomarkers are usually quantitative measures, which can be utilized in diagnosis and monitoring of response to treatment. They are thus crucial to the development of therapies and may be utilized as surrogate endpoints in Phase II clinical trials. To date, there is no specific drug treatment for acute brain injury, and many seemingly promising agents emerging from pre-clinical animal models have failed in clinical trials. Large Phase III studies of clinical outcomes are costly, consuming time and resources. It is therefore important that adequate Phase II clinical studies with informative surrogate endpoints are performed employing appropriate biomarkers. In this article, we review some of the available systemic, local, and imaging biomarkers and technologies relevant in acute brain injury patients, and highlight gaps in the current state of knowledge.

## Introduction

The ictus–the catastrophic event of severe acute brain injury–is followed over the ensuing hours and days by a secondary cascade of pathological processes that can exacerbate neuronal injury and worsen outcome. These secondary changes are potential targets for therapy. Although progress has been made, many patients still experience varying degrees of long-term disabilities post-injury with consequent demands on carers and resources, and there is room for improvement (1).

Significant advances in treatment over the past two decades for traumatic brain injury (TBI) and subarachnoid hemorrhage (SAH) has led to more patients surviving with better outcomes, due to a combination of factors. These include evolution of evidence-based guidelines for TBI management (2), growing recognition of the benefits of managing severe TBI patients in neuroscience centers and procedures for safe transfer of patients to such centers, better triage and assessment of patients on admission, including computed tomography (CT) scans, better neuromonitoring in the neurocritical care unit, both continuously at the bedside [e.g., intracranial pressure (ICP) and brain tissue oxygen monitoring] and intermittently in scans [e.g., CT and magnetic resonance imaging (MRI)], evolution and refinement of protocol-driven therapy for better management of patients, and improvements in surgical procedures and rehabilitation (1). Even so, TBI remains the leading cause of mortality and disability in young people in high-income countries (3). Moreover, TBI is rising in developing countries largely due to wider use of motor vehicles, while a shift toward older age of patients with TBI due to falls is occurring, especially in high-income countries (3, 4). While SAH can sometimes result from trauma (head injury), it can also occur non-traumatically, usually as a result of rupture of intracranial aneurysms in about 80–85% of non-traumatic SAH (5, 6). Aneurysmal SAH carries a high risk of complications, a high degree of dependency in survivors, and most SAH patients are under 60 years of age (5, 6).

Biomarkers are a key aspect of neuromonitoring. A broad definition of a biomarker is any observable feature that can be used to inform on the state of the patient, e.g., a molecular species, a feature on a scan, or a monitoring characteristic, e.g., cerebrovascular pressure reactivity index (PRx). Examples are tabulated (Table 1). Biomarkers are usually quantitative measures, which can be utilized in diagnosis, prognosis, monitoring evolution of injury and recovery, and response to treatments (7). They are thus crucial to the development of therapies in many pathological states and may be utilized as surrogate endpoints in Phase II clinical trials. To date, there is no specific drug treatment for acute brain injury, and many seemingly promising agents emerging from pre-clinical animal models have failed in clinical trials (7). Part of the discrepancy may be due to the much greater heterogeneity of clinical TBI compared with the highly standardized conditions of experimental models (7). To have enough statistical power, Phase III randomized controlled trials of clinical outcomes require large numbers of patients and are costly, consuming much time and resources. Moreover, they may divert crucial numbers of patients away from other clinical trials. Before deciding to embark on Phase III ventures, it is important that adequate Phase II clinical studies with informative surrogate endpoints are performed employing appropriate biomarkers. In this article, we review some of the available systemic, local, and imaging biomarkers and technologies relevant in acute brain injury patients, and highlight gaps in the current state of knowledge.

**Table 1:** **Examples of biomarker methodology in human brain**.

Biomarkers in human brain	Technique	Extent of measurement	Timeframe (and frequency)	Invasive?
Intracranial dynamics: ICP, CPP, ABP, PRx, PbtO_2_ (8–10)	Sensors for pressure and O_2_ concentration	ICP–global PbtO_2_–regional/focal	100–0.1 Hz. Multi-day. Often expressed averaged over time (e.g., hourly)	Yes (insertion of probes into brain)
		
Net changes (import or export) by brain for glucose and lactate (11)	Arteriovenous difference	Global	Multi-day (sampling twice daily)	Yes (insertion of arterial line and jugular venous catheter)
Brain extra-cellular concentrations of small molecules (e.g., glucose, lactate, pyruvate, glutamate, and glycerol) (8)	Microdialysis	Focal	Multi-day (hourly vial changes)	Yes (insertion of catheter into brain)
Regional cerebral metabolic rate of glucose (CMRglc) or oxygen (CMRO_2_) (12, 13)	PET	Global and regional	Usually single scan session (<1 h), sometimes repeated after a few days	Yes (i.v. injection of radioactivity with short half-life)
Cerebral inflammation: presence of activated microglia (ligand PK11195) (14, 15)	PET	Global and regional	Usually single scan session (<1 h), sometimes repeated after a few days	Yes (i.v. injection of radioactivity with short half-life)
Brain extra-cellular proteins < 100 kDa (e.g., cytokines and chemokines) (16)	Microdialysis	Focal	Multi-day (hourly vial changes)–usually several hours pooled (e.g., 4–8 × 1 h vials) for analysis	Yes (insertion of catheter into brain)
Small molecules in brain tissue (e.g., NAA, creatine, choline, myo-inositol, glutamate and glutamine, GABA, lactate) (17)	^1^H-MRS	Regional and voxel	Usually single scan session (<1 h), sometimes repeated after a few days	No
Phosphorus-containing small molecules in brain tissue (e.g., ATP, phosphocreatine, inorganic phosphate) and brain intracellular pH (18)	^31^P MRS	Regional and voxel	Usually single scan session (<1 h), sometimes repeated after a few days	No
^13^C-labeling in metabolites in brain tissue (e.g., glutamate and glutamine) for calculating TCA cycle rate, other ^13^C-labeled species also detectable (e.g., GABA, aspartate, NAA) (19)	^13^C MRS	Regional and voxel	Usually single scan session (ca. 2 h)	Moderately (i.v. bolus and infusion of stable-isotope ^13^C-labeled substrate, e.g., glucose)
^13^C-labeling patterns in metabolites detected extracellularly (e.g., glutamine or lactate) diagnostic for biochemical pathways such as TCA cycle, glycolysis, PPP (20, 21)	^13^C-labeled microdialysis	Focal	Typically 24 h (24 × 1 h vials pooled)	Yes (insertion of catheter into brain, perfused with solution of stable-isotope ^13^C-labeled substrate, e.g., glucose, lactate, or acetate)

*Table adapted from Jalloh et al. (22)*.

*ABP, arterial blood pressure; ATP, adenosine triphosphate; CMR_glc_, cerebral metabolic rate of glucose; CMRO_2_, cerebral metabolic rate of oxygen; CPP, cerebral perfusion pressure; GABA, gamma-aminobutyric acid; ICP, intracranial pressure; i.v., intravenous; MRS, magnetic resonance spectroscopy; NAA, *N*-acetylaspartate; PbtO_2_, brain tissue oxygen concentration; PET, positron emission tomography; PPP, pentose phosphate pathway; PRx, pressure reactivity index*.

## Biomarkers of Intracranial Dynamics

**Figure 1:**
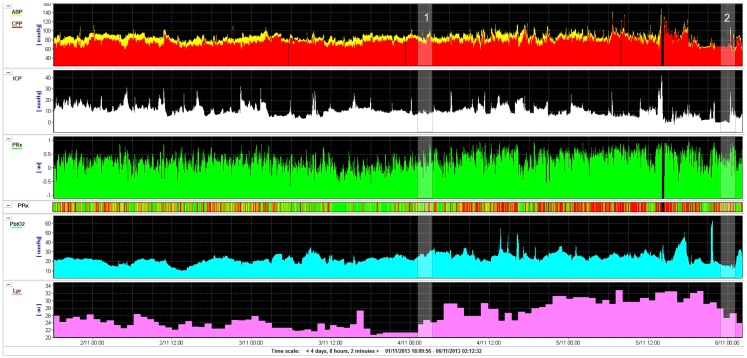
**Example of monitoring (for 4 days) in a road traffic accident victim (aged 22 years): intracranial pressure (ICP), cerebral perfusion pressure (CPP), pressure reactivity index (PRx), brain tissue oxygenation (PbtO_2_), and lactate/pyruvate ratio (LPR)**. Initial GCS was 13 and deteriorated quickly to 7. Patient had never daily averaged ICP above 20 mmHg, CPP was above 70 mmHg all the time. On day 2 after injury, pressure reactivity (PRx) deteriorated (marker 1, this can be also read by “risk plot,” where PRx is color coded: green–good reactivity, red–deteriorated reactivity) and lactate/pyruvate ratio exceeded 25 and 30 later. On day 4, the patient improved (good CT scan), lactate/pyruvate ratio decreased, and PRx improved (marker 2). The patient was weaned uneventfully from the ventilator and successfully extubated shortly afterward. This example shows that lactate/pyruvate ratio and PRx may be markers of deterioration, adding clinical information independently of ICP and CPP.

Raised ICP or inadequate cerebral perfusion pressure [CPP, which is calculated as mean arterial blood pressure (ABP) minus ICP] is one of the most frequent cases of secondary ischemic brain insults after TBI. Example traces are shown in Figure 1. Absolute thresholds for intracranial hypertension and hypoperfusion are known, and they are based on statistical evaluation of large samples of patients, but most probably they are individual- and time-dependent. “Optimal CPP strategy” (23) indicated a possible solution for individualization of CPP management target, using continuous assessment of cerebrovascular reactivity (24, 25). This can be translated to the concept of “optimal mean arterial pressure” in clinical scenarios where raised ICP is not a meaningful factor, e.g., cardiopulmonary bypass surgery (26) and in care of premature newborns (27).

In TBI, monitoring and manipulation of ICP and CPP comprise a cornerstone of intensive care. Often, the same principles of management are extrapolated to other, non-traumatic, acute brain injuries where intracranial compliance and pressure-flow dynamics may be compromised. Despite the lack of level-1 evidence, the Brain Trauma Foundation (BTF) guidelines advocate the implementation of therapies when ICP exceeds the range of 20–25 mmHg and to maintain a CPP in the range of 50–70 mmHg (with a recommended goal of 60 mmHg) (28).

Recognition of the strong association between intracranial hypertension and systemic hypotension with increased morbidity and mortality in patients with TBI has been established, based among others, on landmark studies from the Traumatic Coma Data Bank (29).

The goal of CPP ≥ 70 mmHg or the practice of “indiscriminate” CPP augmentation fell out of favor after a demonstration of lack of clinical outcome benefit coupled with an increased incidence of complications and particularly the acute respiratory distress syndrome (ARDS). Yet another aspect of the CPP debate is the Lund concept or the “ICP and microperfusion-guided management” as proposed by the University of Lund, Sweden (30). Proponents of this strategy focus on ICP control (rather than CPP), maintenance of intravascular volume with the use of colloids, and accept a lower CPP to avoid exacerbating cerebral edema by an increase of water leak through capillaries’ walls into brain.

Intracranial hypertension has been closely linked to adverse outcomes after; nevertheless there remain no large randomized trials that directly compare ICP treatment thresholds. Data from observational studies and non-controlled series have suggested thresholds ranging from 20 to 25 mmHg. The BTF’s latest guideline statement has identified a lack of level-1 evidence and offered, as a level-2, the treatment threshold of 20 mmHg based mainly on the largest available, prospective, observational study (31). In the same document, it is recognized that rather than accepting a generic, absolute ICP threshold, an attempt should be made to individualize thresholds based on patient characteristics, other critical parameters, and on a risk-benefit consideration of treating ICP values (32). Similar to individual target in CPP-oriented therapy, we can define patient-specific, pressure reactivity-guided ICP thresholds by graphing the relationship between ICP and PRx (32). The PRx is a moving correlation coefficient of ICP and mean ABP (mABP), and reflects status of cerebral vascular autoregulation. Positive PRx values imply that increases in mABP are positively associated with increases in ICP, suggesting autoregulatory impairment, whereas negative PRx values indicate preserved vascular reactivity, with vasoconstriction and decrease in ICP in relation to surges in mABP. The PRx has been previously validated against other indices of cerebral autoregulation (25), and is related to outcome (10). PRx may be averaged over time, but also across different CPP levels, giving “optimal CPP” values, and across ICP, giving individual “ICP critical thresholds.”

Another, unexplored concept is to use biochemical markers to establish individual thresholds for rising ICP and falling CPP. Biochemical markers of raised ICP and decreased CPP are primary markers of cerebral ischemia. Most established is the lactate/pyruvate ratio (LPR) measured in brain microdialysates. Timofeev et al. found that LPR, glucose, ICP, and PRx are independent and significant predictors of mortality [along with age and admission Glasgow coma scale score (GCS)] (8). Examples of LPR deterioration that are coincidental with worsening of PRx, but not explained by rising ICP or falling CPP are presented in Figure 1. In a swine model of intracranial hemorrhage (ICH), PRx correlated with LPR and glutamate level (33). Glycerol and LPR associated with pressure reactivity only in perilesional tissue after TBI (9). A spontaneous ICH study (34) has suggested similar association. Both PRx and LPR show time-dependent improvement after severe head injury–on average they both recover after 5 days (35).

Serum and cerebrospinal fluid (CSF) content of IL-8 and, to a lesser extent, tumor necrosis factor (TNF)-α demonstrated the most promise to be candidate serum markers of impending intracranial hypertension and cerebral hypoperfusion after TBI with proteomic mapping (36). In different analysis, S-100β levels in CSF were associated with high ICP and low CPP over a full week of ICP monitoring (37). Other neuroscientists suggested that serum IL-6 can be used for the differential diagnosis of elevated ICP in isolated TBI (38). Initial serum ceruloplasmin/copper levels may have diagnostic value in predicting patients at risk for developing high ICP after TBI (39). Analysis of serum retinol binding protein 4 levels at 24–36 h post-injury indicates it may predict subsequent increases in ICP with a sensitivity of 86% and specificity of 88% at 11.6 μg/mL (40). In this study, the authors have identified 31 candidate biomarkers whose serum abundance was altered after injury.

Intracranial dynamics biomarkers are important for monitoring not only TBI but also SAH (see later Section entitled “Biomarkers of Cerebral Circulation in SAH”).

With further regard to TBI patient management, results were published in 2012 from a randomized trial (BEST TRIP) that enrolled 324 patients with severe TBI in South America, which examined the clinical effectiveness of care using ICP monitoring versus imaging and clinical examination-based monitoring (41). The trial concluded that care focused on maintaining ICP at 20 mmHg or less was not superior to care based on imaging and clinical examination. However, it is important to note that patients in both arms received tiered ICP-lowering therapies, and a decompressive craniectomy was performed in 30% of patients in each arm. Hence, the trial findings do not challenge the belief that brain edema and raised ICP should be actively managed after TBI.

In 2014, a consensus statement on the indications for ICP monitoring has been published (42). This was designed as an interim replacement for the BTF guidelines, as numerous specialists regarded the latter as not being explicit enough. Overall, current clinical practice seems to accept the value of post-operative ICP monitoring in severely head-injured patients.

## Extra-Cellular Biomarkers of Brain Chemistry

### Microdialysis biomarkers of energy metabolism

The pathophysiology of TBI includes derangements to energy metabolism, which are independent of ischemia and evolve over the scale of hours and days (8, 43). Experimental studies suggest an acute increase in glucose metabolism, which relates to disturbances in ionic and neurochemical gradients (44–46). Clinical TBI studies demonstrate changes to glucose uptake and its subsequent metabolism (47–49). The degree of metabolic perturbation observed after injury is affected by interventions and relates to clinical outcome (8, 13, 50–53). Hence, biomarkers of energy metabolism provide important information on the state of the brain-injured patient.

Microdialysis, which allows the *in vivo* sampling of molecules in the extra-cellular fluid of the human brain, is well suited to following energy metabolism. Early microdialysis studies in human brain were conducted by Hillered et al. in patients undergoing operations for brain tumors (54). Studies by Persson et al. pioneered microdialysis in TBI and SAH patients, where ICP was measured in parallel with microdialysate levels of lactate, pyruvate (and LPR), hypoxanthine, glutamate, and several other amino acids (55). These analyses were by high performance liquid chromatography (HPLC). Nowadays, clinical microdialysis analyzers (which utilize enzymatic colorimetric assays on a micro-scale) are commercially available for use at the bedside, and the analytes that they measure, related to energy metabolism, include glucose, lactate, pyruvate, glutamate, and glycerol. The brain is reliant on a continuous source of glucose as an energy substrate, which it uses almost exclusively under normal physiological conditions. Inside the cytosol, glucose undergoes glycolysis to pyruvate, which can be exported into the extra-cellular space and measured with microdialysis, undergo conversion to acetyl-CoA and subsequent metabolism via the mitochondrial tricarboxylic acid cycle (TCA cycle), or be converted to lactate by the action of lactate dehydrogenase (LDH). The TCA cycle results in the recycling of nicotine adenine dinucleotide (oxidized form; NAD^+^) to nicotine adenine dinucleotide (reduced form; NADH). The donation of electrons by NADH is the main driver of mitochondrial electron transport chains and hence, adenosine triphosphate (ATP) generation. Lactate is generated from pyruvate by the action of LDH. Importantly, this reaction also converts NADH to NAD^+^. Normally, cells maintain a tight balance between NAD^+^ and NADH, the cellular redox status. Hence, the balance of lactate and pyruvate in the extra-cellular fluid, the LPR, reflects the redox status inside the cell which in turn reflects the balance between non-oxygen consuming glycolytic metabolism and oxidative mitochondrial metabolism. The LPR increases if oxidative mitochondrial metabolism reduces due to either hypoxia and/or mitochondrial damage. Glutamate and glycerol are indirectly related to energy metabolism, as both are biosynthesized from products of glucose metabolism. In the context of brain injury, observed extra-cellular increases in glutamate and glycerol are thought to reflect excitotoxicity and cell membrane breakdown, respectively.

Temporal changes in microdialysate glucose concentration might serve a useful biomarker after brain injury. Microdialysate glucose concentrations reflect the balance between glucose delivery and utilization; a fall in concentration indicates reduced delivery and/or increased utilization. Several studies have found an association between glucose concentrations and outcome with averaged glucose concentrations lower in those patients with poor outcome after SAH or TBI (56–58). In the largest study to date of microdialysis in TBI patients, a multivariate logistic regression model found that total monitoring averaged glucose was actually a positive predictor of mortality such that higher averaged glucose concentrations predicted death (8). Regarding temporal trends in individual patients’ glucose concentrations, Vespa et al. observed three patterns of daily mean glucose in 30 patients with severe TBI (59). Patients with a good outcome were found to have initially normal glucose concentrations (0.5–1 mmol/L) that later declined, as opposed to glucose concentrations that were low or variable from the start of monitoring (59). Other authors have also observed patterns of declining glucose in brain-injured patients with time (8, 35).

The situation for glucose concentrations versus clinical outcome is thus complex and still remains poorly understood. A likely explanation is that there may be an optimal range of brain extra-cellular glucose concentrations, and that levels below and above this range are detrimental. Moreover, circulating glucose levels in blood presumably influences brain extra-cellular glucose concentrations. While optimal glucose ranges have yet to be established with certainty, existing evidence, albeit from a small study, suggests that blood glucose levels within the range of 6–9 mM and brain microdialysate glucose levels of 1–5 mM were associated with minimizing the corresponding brain microdialysate LPR and glutamate concentration, while microdialysate glucose levels <1 and >5 mM were associated with high LPR and high glutamate, respectively (60). Firmer evidence for optimal glucose ranges may emerge from the large datasets that continue to accrue in major centers, as well as from more specialized studies involving labeled glucose and scanning technologies.

Elevated lactate and an elevated LPR have both been associated with worse outcomes after both TBI and SAH (8, 57, 58, 61–63). However, there is again not a straightforward linear relationship between the concentration of lactate, the adequacy of energy metabolism and clinical outcomes. Increasingly recognized is the use of lactate as an energy substrate by the brain particularly after injury (11, 64). Hence, the extra-cellular concentration of lactate reflects both the balance between substrate delivery and utilization, and increased generation due to hypoxia and/or a shift in metabolism away from mitochondria (11, 61). The LPR perhaps better represents the energy status of cells and has been found in several studies to discriminate between outcome groups (8, 56, 63, 65).

Glutamate concentrations that increase over the course of the monitoring period are associated with worse outcomes in TBI patients (66). Glutamate concentrations have also been associated with the development of delayed ischemic neurological deficits (DIND) after SAH (67, 68) and outcome at 12 months after SAH (69). Similarly, some studies have found an association between glycerol and outcome (70, 71).

Individual microdialysis parameters are not 100% specific for derangements in energy metabolism; different combinations of microdialysate markers have been used in an attempt to better predict clinical course and outcomes. In poor-grade SAH patients, a combination of a LPR > 40 and glucose < 0.7 mmol/L was associated with death or severe disability using logistic regression (72). In severe TBI patients, the combination of a LPR > 25 and glucose < 0.8 mmol/L was a strong predictor of poor outcome using a multivariate model (73). Microdialysis parameters have also been combined with other physiological measures, such as brain tissue oxygen, to improve their predictive power (74).

There are some important limitations of using microdialysis to follow energy biomarkers after brain injury. Firstly, microdialysis is an invasive technique and so limited to those patients suffering a severe brain injury but not those with milder forms of injury. Secondly, microdialysis data can show a high degree of both between and within patient variability, which is masked by studies that use averaged data. Nelson et al. found microdialysis markers in 90 TBI patients to be highly auto-correlated such that subject identity alone (and not CPP, ICP, or outcome) explained 52-75% of the variance in microdialysis data (75). Thirdly, microdialysis allows the sampling of small extra-cellular molecules up to 100 kDa in size and so any larger biomarkers of energy metabolism would not be detected.

Complementary to extra-cellular measures of brain chemistry is *in vivo* magnetic resonance spectroscopy (MRS), which measures brain tissue chemistry (see later section entitled In vivo Biomarkers of Brain Chemistry).

### Serum biomarkers of energy metabolism

Tracking cerebral energy metabolism by measuring substances outside of the central nervous system (CNS) is challenging due to the non-specific nature of the substrates and enzymes involved in energy metabolism and due to the BBB limiting substances passing from the brain to the circulation. One substance that has been identified as a marker of brain injury and which relates to cerebral energy metabolism is neuron-specific enolase (NSE). Enolase is one of the enzymes essential for glycolysis, catalyzing the conversion of 2-phosphoglycerate to phosphoenolpyruvate. Several enolase isoforms have been identified including NSE, which is thought to localize specifically to the cytoplasm of neurons and has attracted interest as a potential serum biomarker for brain injury. Several studies have demonstrated the value of serum or CSF NSE levels in predicting pathological changes on head CT, although NSE levels are not as specific or as sensitive as S-100 calcium binding protein B [S-100B, a multifunctional protein produced mainly by astrocytes (76)] or glial fibrillary acidic protein [GFAP, an cytoskeletal intermediate filament protein with multiple functions (77)], evaluated as markers in TBI patients’ serum (78–81).

In terms of brain energy supply, serum glucose concentration has received the most attention. For example, in a database of 327 TBI patients‘ admission blood parameters, hierarchical log linear analysis revealed that age, raised serum glucose (>7.1 mmol/L), low hemoglobin, and GCS, each had a direct independent statistical relationship with clinical outcome (82). Control of serum glucose during on-going neurocritical care is achieved using insulin, and there has been debate about whether tight or looser glycemic control is better. Recent opinion is in favor of the looser control, as exemplified by the findings of a detailed crossover study in 13 TBI patients monitored by ^18^F-fluorodeoxyglucose (FDG)-positron emission tomography (PET) scans and brain microdialysis (83). In that study, there were more frequent critical reductions in microdialysate glucose and elevations of microdialysate LPR during tight glycemic control. We have already mentioned (above) another small study in which blood glucose levels of 6–9 mM and brain microdialysate glucose levels of 1–5 mM were associated with optimal brain microdialysate chemistry (60). While the underlying mechanisms interlinking serum glucose with brain chemistry and physiology are still not fully understood, a very recent study in 86 TBI patients has indicated an interesting association between increasing serum glucose levels and increasing cerebrovascular PRx (84). The issue of management of blood glucose levels after brain injury, and the twin problems of hypo- and hyper-glycemia have been highlighted in a recent review (85). Besides glucose, recent evidence has also suggested that circulating lactate may be able to play a role as an alternative or supplementary source of fuel for the human brain, see for example TBI patient studies of endogenous lactate (11) and exogenous lactate intravenous administration (64). However, this is a topic of much controversy (86, 87).

## Biomarkers of Brain Structural Imaging

Computed tomography is the imaging modality of choice in the acutely unwell injured brain due to its speed and relative ease of imaging critically ill patients, particularly with the advent of mobile machines which allow for imaging within the intensive care unit itself. However, while useful in the detection of cerebral edema, blood, and large lesions, it is clear that CT greatly underestimates the extent and distribution of injury after brain injury and correlates poorly with outcome. Issues including the requirements for MRI compatible equipment, and the logistical issues of transporting critically unwell patients to the MRI suite mean that CT is likely to remain the modality of choice in the acute phase. However, in contrast to many of the biomarkers used in neurointensive care, MRI offers a unique opportunity for *in vivo* assessment of the entire brain.

**Figure 2:**
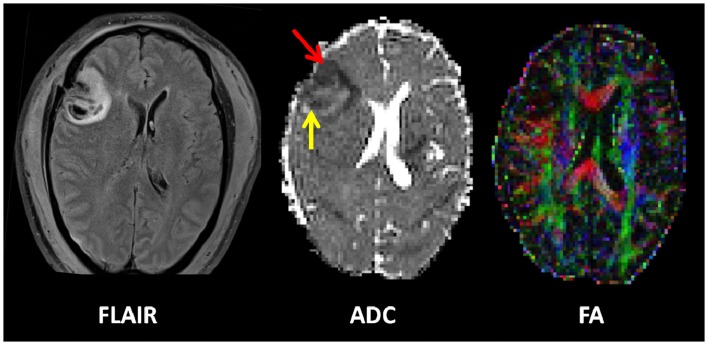
**Example of an early contusion in a 49-year-old male who sustained a severe TBI after an alleged assault**. He was GCS 4 at the scene. Imaging was performed approximately 48 h after injury. A left frontal contusion can be clearly seen on the FLAIR image. The apparent diffusion coefficient map (ADC) shows a cytotoxic rim (red arrow) and vasogenic rim (yellow arrow). The combined fractional anisotropy and directional map (FA) shows loss of fibers integrity at the site of the contusion. Color hue indicates direction as follows; red, left–right; green, anteroposterior; blue, superior–inferior.

Magnetic resonance imaging is increasingly being used in the context of TBI. Examples are shown in Figure 2. Conventional MRI sequences (which include T1- and T2-weighted, fluid-attenuated inversion recovery (FLAIR; good for the detection of edema) and gradient recalled echo (GRE; for the detection of blood products) have been clearly shown to be better than CT for the detection of lesions, particularly traumatic axonal injury (TAI), posterior fossa lesions, and brainstem lesions (88). Yet despite improved lesion detection, many patients, particularly at the mild end of the spectrum, have no abnormalities seen on CT or conventional MRI, but still experience significant neurocognitive sequelae following TBI. Even in the more severely injured patients, the distribution of lesions detected are also often not sufficient to explain the deficits seen. Persistent symptoms may be the result of subtle microstructural alterations, such as TAI, which is often demonstrated at post-mortem neuropathology, which lie below the threshold of detection and are hence not demonstrated by conventional imaging during the patient’s life (89, 90).

Despite the complexity, *in vivo* characterization as offered by advanced MRI techniques offers the potential not only for improved detection of the extent and distribution of injury but also may allow for a unique *in vivo* window into some of the pathophysiological processes that occur after TBI. Diffusion tensor imaging (DTI) offers the most promise to help disentangle the complexities of the anatomical basis of neurotrauma, and has been shown to allow the exquisite delineation of the burden of injury, particularly TAI. The diffusion tensor characterizes the magnitude of water diffusion [apparent diffusion coefficient (ADC)], its directional non-uniformity (anisotropy), and its orientation (eigenvalues). The exact mechanisms of the nature of water diffusion in both gray and white matter are incompletely understood, however the organization of tissue structure including the presence of myelin, microtubules, and organelles, as well as the contribution of intra- and extra-cellular water play a significant role. Susceptibility-weighted imaging (SWI) (see below) is increasingly being used as it is exquisitely sensitive to the presence of blood products and improved the detection of micro-hemorrhages secondary to shearing forces.

It is becoming apparent that the timing of MRI is important after brain injury, particularly when being used as a prognostic biomarker. In moderate-to-severe TBI, the number and volume of lesions detected on FLAIR, gradient echo (GE), and diffusion weighted imaging (DWI) decreased over time in patients imaged at a median of 7 days, 3 months, and 12 months (91). The number of DWI lesions and FLAIR lesion volume in the early MRI were associated with outcome, indicating that early MRI is important for questions of prognosis. A multi-national study [Neuro Imaging for Coma, Emergence Recovery, Consortium (NICER)] investigating DTI parameters in patients who remain in a coma after the first week of intensive care, shows promise for the prediction of poor versus good outcome (92).

There are relatively few studies in humans investigating the hyper-acute (within 72 h) and acute phases of TBI. In patients with moderate-to-severe head injury imaged at a median of 32 h after injury, fractional anisotropy (FA) was decreased in the white matter predominantly as a result of increased radial diffusivity consistent with edema (93). In patients with contusions, three distinct regions are seen within the first 72 h; a core of restricted diffusion, surrounded by an area of raised ADC (likely vasogenic edema) and a thinner rim of reduced ADC (likely cytotoxic edema), which is subsumed by the vasogenic rim after 72 h. This outer cytotoxic rim may represent a “traumatic penumbra” that may be rescued by effective therapy.

Imaging performed in the sub-acute or chronic stages after TBI typically find consistent reductions in FA in classical areas affected by TAI, even when conventional MRI shows no lesion. These regions include the sub-cortical white matter in the frontal and temporal regions, splenium of the corpus callosum, posterior limb of the internal capsule, the cerebral peduncles, and whole brain white matter. A recent meta-analysis concentrating on mild TBI studies by Aoki and colleagues found a decrease in FA and increase in ADC in the corpus callosum (and in particular the splenium) (94). The global nature of abnormality found in what often appears to be structurally normal appearing tissue is underpinned by a study that relates DTI abnormality to the full spectrum of outcome, ranging from the vegetative state to those with minimal or no disability. Here DTI abnormalities in a broad range of regions were noted to scale with clinical outcome (95). A more detailed discussion of the reported DTI literature in TBI is provided in a review by Hulkower and colleagues (96).

A relatively recent MRI development is SWI, which shows promise for imaging the cerebral vasculature, micro-bleeds, iron deposition, and calcification. SWI has been applied clinically in TBI and other brain pathologies (97, 98). While SWI does not replace conventional MRI modalities, it can provide potentially valuable complementary information that moreover may prove useful in clinical interventional trials (97, 98).

It is clear that dynamic changes able to be detected by imaging are not confined to the hyper-acute stage (99, 100). Using advanced MRI techniques to improve our knowledge of longitudinal patterns of change in patient populations is important. Not only will it aid the interpretation of imaging findings in individuals, but may also provide further insight into late pathophysiology, help select appropriate patients for clinical trials, and provide a framework that allows MRI to be used as an imaging biomarker of therapy response.

In comparison to TBI, there has been relatively little research in the use of MRI as a biomarker after SAH. In part this may part be secondary to the technical difficulties of performing MRI in patients with extensive extra-vascular blood products and the need to ensure that any clips and coils are MRI safe. The presence of such clips causes artifacts that may impact image quality (101). A recent study has found cerebral micro-bleeds (on T2* GE) related to the presence of DWI lesions in almost 46% of patients imaged within 7 days of ictus (102). The clinical significance of such lesions is unknown. In poor-grade SAH patients, DWI within 96 h of ictus multifocal areas of ischemia can be seen (103). In patients who do not experience re-rupture, the presence of parenchymal lesions with 24 h was associated with poorer outcome (104). However, the numbers of patients looked at with such techniques are small and much larger studies are required. Diffusion-perfusion mismatch may be seen (105, 106). However, it may be technically difficult in the presence of large amounts of blood products and it has not been compared to other methods including transcranial Doppler (TCD) ultrasonography or Perfusion CT.

Magnetic resonance imaging, including advanced sequences, clearly has much to offer in the detection of injury, the prediction of outcome, and greater understanding of the pathophysiology processes that occur after a brain injury. It is likely that it will be increasingly used in the acute and sub-acute phases of injury to help answer key clinical questions.

## *In vivo* Biomarkers of Brain Chemistry

**Figure 3:**
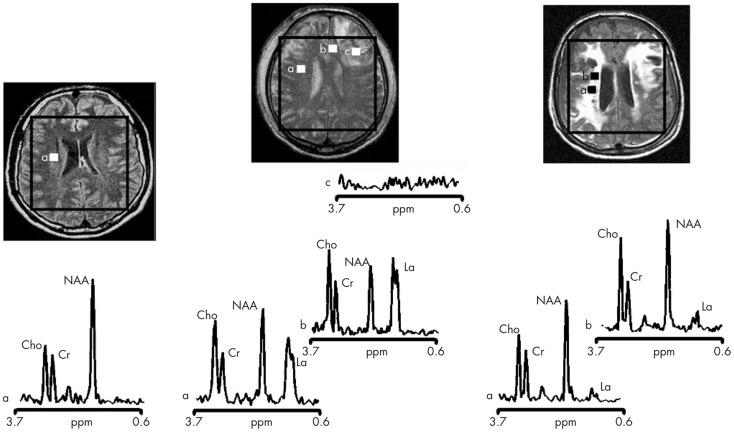
**Proton magnetic resonance spectra and conventional magnetic resonance images showing the volume of interest for spectroscopic imaging of a normal control (left panel), Patient 1 (central panel), and Patient 8 (right panel) with traumatic brain injury (TBI) (**17). On conventional MRI, Patient 1 shows a focal hematoma in the frontal left hemisphere and Patient 8 shows diffuse MRI abnormalities. Spectra show decreases of *N*-acetylaspartate (NAA) and increases of choline (Cho) and lactate (La) in patients with TBI (a and b in central and right panels) with respect to the normal control (a in left panel). The spectra of Patient 1 (central panel) show more pronounced metabolic abnormalities than those of Patient 8 (right panel), despite the fact that Patient 8 showed markedly more abnormalities on conventional MRI. In the spectra of Patient 1 (central panel), metabolic abnormalities are clearly evident in the normal appearing brain. Finally, in Patient 1, voxels inside the focal hematoma (c in central panel) were excluded to avoid the artifacts that could be derived by the cerebral hemorrhagic contusion. Cr, creatine [Reproduced from J Neurol Neurosurg Psychiatry, Marino S, et al. 78:501–7 (2007) with permission from BMJ Publishing Group Ltd (17)].

Magnetic resonance spectroscopy *in vivo* can be used to explore cellular metabolic status and evidence of cellular injury and so may provide important insights into the complex biochemical and pathophysiological processes after TBI. However, it can be technically difficult to obtain robust spectra and it is only relatively recently that whole brain techniques have become available (107). The majority of studies have studied mild TBI and/or long-term injuries and used a limited number of (or single) voxels, using ^1^H-MRS. Within 8 h of injury a reduction of *N*-acetylaspartate (NAA) is apparent (108), and may return to normal levels in patients who make a good recovery (109). In 10 moderate-to-severe TBI patients imaged 48–72 h post-ictus, NAA (a marker of neuronal damage) ratios were found to be decreased and in 5 patients lactate was increased (17) (see Figure 3 for examples). The NAA and lactate levels correlated with GCS at presentation and 3 months Glasgow outcome scale score (GOS) and which suggests early imaging may be important for prediction. Preliminary studies suggest that ^1^H-MRS may also be informative about outcomes, with studies finding it may discriminate between those who die or remain in a vegetative state compared to those who recover awareness (110–112). However, the best timing of scans, and optimal positioning of spectroscopy voxels (if whole brain methods are not used) is not yet determined. A more detailed consideration of the aspects of brain chemistry that can be addressed by ^1^H-MRS as well as by ^31^P and ^13^C MRS is detailed below.

### Practical considerations for acquisition of *in vivo* spectra

Besides providing images of the brain, magnetic resonance enables a chemical signature (spectrum) to be determined for the regions of interest (ROI). The nuclei that can be addressed by MRS at the endogenous level (without administering exogenous label) are ^1^H and ^31^P. These spectra can be obtained with either a surface coil or head-coil. ^31^P MRS can be performed with or without ^1^H decoupling, and the ^1^H channel of the coil provides “scout images” for visualizing brain anatomy and setting voxels (3-dimensional grid) for selection of ROIs. MRS is achievable for endogenous ^1^H and ^31^P because these nuclei possess high natural abundance (99.985 and 100%, respectively). In contrast, ^13^C, the magnetic resonance-responsive isotope of carbon, has a natural abundance of only 1.1%, while the most abundant carbon isotope is ^12^C (98.9%) that is not itself magnetic resonance-responsive. Therefore, ^13^C MRS requires large amounts of ^13^C-labeled substrates to be infused intravenously or given orally. Also, ^13^C MRS is currently limited to surface coils, because of the radio-frequency power absorption by the brain as a consequence of decoupling ^1^H from the ^13^C to achieve an interpretable ^13^C spectrum. The specific absorption rate (SAR) limit to minimize heating of tissue is especially critical with regard to the eyes and so ^13^C MRS is done using surface coils to address ROIs of the brain avoiding the eyes. Any future design of ^13^C MRS head-coils would thus necessitate adopting a partial head-coil architecture avoiding the eyes.

**Figure 4:**
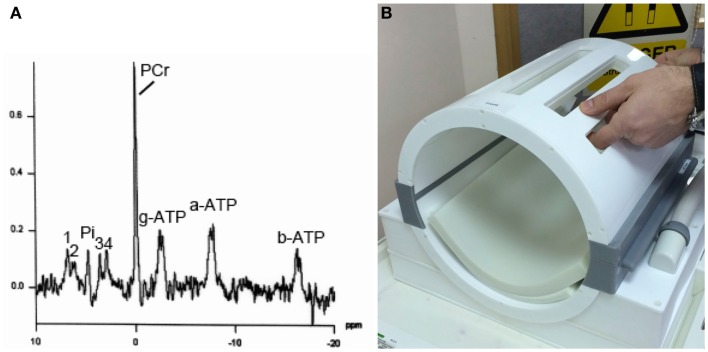
**(A)** Example of ^31^P MRS in brain of healthy volunteer (male, 57 years). Signals detected are phosphocreatine (PCR), alpha-, beta-, and gamma-ATP (a-ATP, b-ATP, g-ATP), inorganic phosphate (Pi), phosphatidylethanolamine (1), phosphatidylcholine (2), glycerophosphoethanolamine (3), and glycerophosphocholine (4), in a 3 cm × 3 cm × 3 cm voxel, on a Siemens Verio 3-T MRI scanner using a bespoke PulseTeq birdcage/clamshell ^31^P head-coil illustrated in **(B)**. The head-coil opens up along the joins (gray), designed to facilitate use with patients. Images are courtesy of the Wolfson Brain Imaging Center.

Only a few centers worldwide have neurocritical care units adjacent to MRI scanners equipped for MRS and the expertise to support fully ventilated severe TBI patients through the procedure. Another hurdle to MRI/MRS of such patients is that they often have intracranial monitoring probes, e.g., for ICP and P_bt_O_2_, and in some cases microdialysis. In many cases, MRI/MRS is possible with these probes kept *in situ* but with the connecting leads, tubing and pumps disconnected for the duration of the scan. Such probes have to be tested beforehand (without the patient) with the relevant MR coils, to ensure that they do not produce undue heating (113–115). Another difficulty is the physical size, shape, and location of the intracranial probes themselves, which limits the positioning of surface coils and makes use of conventional “birdcage” head-coils difficult. A way round this problem is a birdcage-clamshell design (e.g., Figure 4) that opens up to facilitate use with patients who have intracranial probes.

Consequent to the above constraints, the majority of human brain MRS data thus comes from healthy adult volunteers and those patients who are sufficiently well to be brought easily to the scanner. These include various brain conditions (e.g., neurodegenerative diseases and psychiatric disorders) and a smaller number with mild TBI. Much of the wider MRS literature focuses on tumors in various organs in cancer patients, and muscle in healthy individuals and patients with “metabolic” diseases (e.g., diabetes).

### Biomarker features of the *in vivo* spectra

The most commonly used form of *in vivo* spectroscopy is ^1^H-MRS (for examples see Figure 3). The most abundant signal in the brain ^1^H spectrum is usually NAA. The peptide *N*-acetylaspartylglutamate (NAAG) is a product of NAA and the two are inter-convertible. NAAG is a small peak in the brain ^1^H-MRS that is difficult to distinguish from NAA. Often both are considered together as “total NAA” that is usually interpreted as a marker for health, viability, and/or number of neurons, particularly their mitochondria. Reduction in NAA is regarded as indicating dysfunction (permanent or temporary) of neuronal tissue. Other ^1^H signals include creatine [combined signal from creatine and phosphocreatine (PCR)], choline-containing molecules, myo-inositol, glutamate, and glutamine (often considered together as Glx as the two species’ signals are incompletely resolved), gamma-aminobutyric acid (GABA), and lactate.

For a molecular species to be detected on ^1^H-MRS, it has to be present at millimole per liter concentrations and also be mobile (i.e., free to tumble rapidly–not bound to or closely confined by membranes or macromolecules). Given these caveats, ^1^H-MRS detects total intra- plus extra-cellular molecules. The intracellular environment dominates the brain volume – it has been estimated that the brain extra-cellular space between cells only comprises about 20% of the total brain volume (116–118). Brain MRS thus appears predominantly to reflect the intracellular compartment and the measures are thus complementary to the extra-cellular measures obtained by microdialysis (see earlier section entitled Extracellular Markers of Brain Chemistry). For example, lactate that is abundant extracellularly (at millimole per liter concentrations) is much less evident on ^1^H-MRS of normal or TBI brain. Moreover, depending on the choice of echo time (TE), these small lactate signals can virtually disappear or appear inverted. Interpretation of lactate is further complicated by overlap with lipid signals. Thus, some ^1^H-MRS studies of normal and TBI brain do not consider lactate at all, e.g., Ref. (119). Although lactate is a normal component of energy metabolism, if lactate appears elevated in a tissue on ^1^H-MRS it is usually a sign of pathology, e.g., in tumors. In TBI, lactate elevation can be seen on ^1^H-MRS in some but not all instances, illustrated in Ref. (17).

Gamma-amino butyric acid, which has very low (usually <1 μmol/L) extra-cellular concentration, shows up as a ^1^H-MRS signal reflecting its much higher intracellular concentration, e.g., 1 mMU (120). MRS-detectable GABA likely reflects cytosolic GABA, as the portion of intracellular GABA that is bound in vesicles is thought to be less readily detectable by ^1^H-MRS, and the same applies to glutamate.

Because of the difficulties in calibrating *in vivo* spectra, ^1^H-MRS results are usually expressed as a ratio, e.g., NAA ratio to creatine. In some studies, absolute concentrations of metabolites have been presented by means of comparing the *in vivo* signals to those of an artificial “phantom” – a large (e.g., 2 L) spherical container of known concentrations of the metabolites of interest in a “physiological” solution, e.g., Ref. (121). Total creatine is generally regarded as a marker of health in all types of brain cells and its levels appear fairly constant in the absence of pathology. However, the creatine signal may increase or decrease in pathological conditions, e.g., ischemic stroke and brain trauma (122, 123). The brain ^1^H-MRS signal from choline is attributed predominantly to free choline, acetylcholine, and cytidine diphosphate choline, which are all “mobile,” rather than the more abundant choline-containing phospholipids that are not freely mobile as they are present in myelin and cell membranes so contribute less signal (124). The ^1^H-MRS signal for choline is interpreted as a measure of cell density and/or rate of membrane turnover or breakdown (124). Myo-inositol interpretation in brain ^1^H-MRS is somewhat debatable, often being regarded as a glial marker but lacking true specificity (124). ^1^H *in vivo* MRS in TBI clinically in patients (Figure 3) and in animal models have suggested NAA as the best biomarker, often expressed as a ratio to creatine, or sometimes to choline (17, 125).

^31^P MRS is less commonly performed than ^1^H-MRS, and requires a dedicated coil (^31^P surface coil or ^31^P head-coil). ^31^P MRS is typically used to measure ATP in tissues, including human brain (Figure 4). While absolute ATP concentration is difficult to measure *in vivo* in human brain, the ratio of PCR to γ-ATP is a well-recognized measure of energy status. A high PCR/ATP ratio is usually interpreted as good energy status of the tissue, with “store species” PCR being well-stocked relative to “active” species ATP. However, somewhat lower PCR/ATP ratios are not necessarily harmful *per se*, but in some cases can be a physiological adaptation, e.g., shown in human heart muscle (126). The ratio of PCR to inorganic phosphate (Pi) is another recognized ratio. Similar to the interpretation of PCR/ATP ratio, a high PCR/Pi ratio is regarded as indicative of good energy status, although the small area under the Pi peak is not easy to measure (integrate) accurately. A more sophisticated ^31^P MRS method of quantifying tissue energetics is measurement of ATP inter-conversion with ADP by ^31^P-magnetization transfer techniques, including *in vivo* saturation transfer (127). This is technically very challenging and, moreover, it has a similar limitation to the simpler PCR/ATP ratio, being unable to ascertain whether the apparently “good” production of ATP is simply due to a lot of glycolysis (“anaerobic” metabolism, probably by glia) that is presumed less favorable than mitochondrial energy metabolism (TCA cycle and oxidative phosphorylation by neurons). Multi-parametric, complementary analytical techniques, in addition to ^31^P-magnetization transfer, are therefore needed to more fully characterize oxidative function and mitochondrial metabolism *in vivo* (127). Much debate still exists on this matter.

Another parameter that ^31^P MRS can measure is tissue intracellular pH, based on the difference in chemical shift (in parts per million) between Pi and PCR (18, 128). Studies of a TBI model, fluid percussion injury in rats, with *in vivo* MRS of brain pre-injury and at a range of time-points up to 8 h post-injury suggest a transient intracellular pH decrease (^31^P), lowest at 40 min then recovering, alongside an initial fall in PCR/Pi ratio (^31^P) at 40 min followed by a temporary rise and subsequent overall decline, and a transient lactate increase (^1^H) peaking at 40 min then subsiding (129, 130). In TBI patients, brain intracellular pH measured by *in vivo* ^31^P MRS was reported as slightly higher than in control subjects (131). The relationship between intra- and extra-cellular brain pH is unclear. Extra-cellular brain tissue pH has been measured in TBI patients using the Neurotrend multi-parameter probe (Codman, Raynham, MA). This study suggested lower extra-cellular brain pH in those TBI patients who ultimately did not survive, compared with ultimate survivors (132). Moreover, occurrence of low extra-cellular pH was a significant discriminator of ultimate mortality in TBI patients, despite normal brain tissue oxygen concentration, in a prospective study (133). Although these results look promising, the Neurotrend sensor is no longer manufactured, and there is currently no commercially available clinical intracranial pH probe. The relationship between intra- and extra-cellular brain pH is thus likely to remain uncharted for the foreseeable future. Nevertheless, there is scope for further investigating use of ^31^P *in vivo* MRS to evaluate brain intracellular pH in TBI patients.

^13^C *in vivo* MRS has been mostly used to evaluate the TCA cycle. The most commonly used substrate is intravenous 1-^13^C-glucose. Besides the glucose itself, the major labeled signals detected are for glutamate plus smaller signals for glutamine (sometimes considered together as Glx) and minor signals for aspartate. Mathematical modeling of the kinetics of glutamate and glutamine labeling enables the TCA cycle flux to be calculated. Other species detectable in ^13^C *in vivo* MRS include GABA, glycerol, myo-inositol, creatine, choline, NAA, lactate, alanine, and bicarbonate. Other ^13^C-labeled substrates that have been employed include 1,6-^13^C_2_ glucose, 2-^13^C glucose, and 2-^13^C acetate (134). The latter is regarded as a glial substrate, whereas glucose is a fuel for both neurons and glia.

Besides directly observed ^13^C MRS, an alternative approach is indirect observation of ^13^C via the ^1^H spectrum, termed ^1^H-observe [^13^C-edited] spectroscopy, or proton-observe carbon edited (POCE) spectroscopy. The advantages of POCE are that ^1^H-MRS is inherently more sensitive than ^13^C MRS and that POCE directly gives ^13^C fractional enrichment values (134). However, a disadvantage is that the indirect (POCE) modality is more complicated to perform and interpret than directly observed ^13^C MRS. Part of the difficulty is that the ^1^H spectrum has a relatively narrow *x*-axis frequency scale (parts per million), and moreover many of the signals are multiplets due to spin couplings with neighboring protons, so the spectrum contains complex, overlapping signals, making accurate de-convolution a challenge.

### *Ex vivo* NMR measurements of brain extra-cellular molecules

High-resolution nuclear magnetic resonance (NMR) spectroscopy can be used *ex vivo* to characterize extra-cellular molecules from the brain, sampled using microdialysis. Characteristics of microdialysis are that it is selective for hydrophilic molecules < 100 kDa the extra-cellular fluid, and is very focal; it can be done with a brain tissue oxygen sensor in proximity, plus an ICP probe.

Microdialysis is good for measuring lactate, which predominantly shows up extracellularly (see section on Energy Biomarkers above), whereas lactate is often difficult to measure in brain tissue using *in vivo* MRS (see section on MRS Biomarkers above). Microdialysate lactate (endogenous) is usually quantified using enzymatic colorimetric assays on a bedside analyzer (CMA600 or ISCUS). Besides lactate, microdialysis can sample other endogenous extra-cellular molecules. Also, labeled substrates (e.g., ^13^C) can be administered directly into the brain extra-cellular space via the microdialysis catheter. Brain cells can take up these substrates, and the metabolites that exit from the cells can be simultaneously collected via the same microdialysis catheter and analyzed by high-resolution NMR in the laboratory (21, 135).

In contrast to the MRS techniques above which are “snapshots” for the duration of the scan, microdialysis is a continuous flow technique. Microdialysates collected are analyzed in suitable sample volumes depending on the assay required, e.g., hourly for endogenous measurements (ISCUS/CMA600), or a 24-h pool for ^13^C-labeling studies (^13^C NMR). In principle, shorter sampling/pooling intervals could be achieved by adopting suitable technology, e.g., a microcryoprobe on NMR. Mass spectrometry could also in theory be used for analyzing smaller (shorter) sampling pools but at the expense of at least some information on intra-molecular positions of label within the metabolite molecules. Intra-molecular positions of label are diagnostic for the metabolic pathways and so NMR is the “gold standard” for deducing metabolic pathways in ^13^C-labeling studies.

## Inflammation Biomarkers

The brain has traditionally been considered as an immunologically privileged site as a result of the blood–brain barrier, absence of a lymphatic system, and lack of peripheral immune surveillance of CNS antigens. However, innate inflammation is a highly conserved process that is involved in all types of pathology and inflammatory mediators such as cytokines and chemokines are increasingly recognized as mechanistic mediators of several CNS pathologies (16, 136). This has led to the investigation of these mediators as possible biomarkers of injury or underlying pathological processes.

A number of cytokines and chemokines have been recovered from blood, CSF, and microdialysate following TBI (16). Several studies have attempted to correlate various clinical parameters with these mediator levels in order to develop their use as biomarkers. Interleukin-1beta (IL-1b) is the prototypic pro-inflammatory cytokines and has been measured in a wide range of cerebral pathologies. In TBI, raised CSF concentrations of IL-1b have been correlated to both increased ICP (137) and worsened outcome (138). IL-1b has also been recovered from the brain extra-cellular space using microdialysis. Moreover, the balance between IL-1b and its endogenous antagonist IL-1 receptor antagonist have also been related to dichotomized clinical outcome (139). In contrast, raised IL-6 concentrations have been related to an improved outcome in several studies (140–142). TNF has also been closely investigated as a cytokine with potentially deleterious effects in animal models; however no relationship could be found between CSF TNF concentrations and either blood–brain barrier permeability (143) or ICP (137, 144). CSF IL-8 has been found to correlate with both increased blood–brain barrier permeability (145) and mortality (146). However, plasma IL-8 was not found to correlate with outcome (147).

Cytokines have also been measured following ischemic stroke. Some studies have found a correlation with outcome such as between plasma IL-6 and stroke severity (148) (increased levels correlate with worse outcome) while others have found raised levels of several plasma mediators such as IL-6, matrix metalloprotease 9 (MMP-9), and S-100B but no relationship to outcome (149).

Taken together the evidence for cytokines as biomarkers is often conflicting and inconsistent. There are several reasons why cytokines may not be easily used as biomarkers. Firstly, the same mediators may behave in a different fashion depending on the exact context in which they are released (150–153). Secondly, there are multiple interactions between cytokines that mean that the cytokine milieu may impact on the action of any given biomarker such that it is overly simplistic to simply classify a mediator as damaging or protective (154). Thirdly, the time course over which a cytokine is released in relation to injury has an important effect on the concentration measured. Variations in sampling time can result in widely differing measured concentrations as the absolute levels can change by several orders of magnitude (16). Finally, because of the complexity of cytokine cascades, many cytokines are highly correlated between themselves making it difficult to infer causality from observational studies, even when a cytokine appears to predict a given clinical outcome (16, 155). Our view would be that, as fundamental mediators of the underlying pathophysiology, cytokine measurements are likely to be important biomarkers, however, a more nuanced approach to univariate correlations is required that takes into account the multiple possible interactions and utilizes panels of cytokines and patterns of expression (155).

Although not yet in widespread use, recent clinical research has demonstrated the principle of using PET with (*R*)-^11^C-PK11195, a ligand for activated microglia, to image neuroinflammation, following up patients in the months and years after TBI (14, 15). As a result, it has been suggested that therapeutic interventions may be beneficial for longer periods after trauma than previously assumed (15). Greater availability of PET technology in future would facilitate the measurement of neuroinflammation and might offer a useful guide to therapy.

## Cell Death Biomarkers

In the hours and days following the primary injury, complex and variable series of changes, including energy perturbations, inflammation, and cell death, occur in the injured brain, leading to further neurological impairment and mortality. Due to its delayed nature, this secondary injury is potentially preventable. A goal of TBI research is development and clinical validation of novel pharmacological treatments to improve outcome. Studies using light and electron microscopy, cell/tissue staining, and DNA fragmentation have been instrumental in illustrating cell death, by apoptosis and necrosis, in cell and animal models of injury. However, they offer only a snapshot of a complex evolving process. Apoptotic cells can die and disappear within hours, so can be missed if the cells or tissue is analyzed too early or too late. The continuous drainage of CSF, often used to manage life-threatening intracranial hypertension in TBI patients, presents an opportunity to measure biomarkers of cell death in the brain of human TBI patients over periods of time. The changing levels of these biomarkers, typically analyzed by ELISA or immunoblotting techniques, has begun to shed light on time-dependent cell death in the brains of TBI patients.

To summarize, biomarkers used to elucidate pathways of cell death in human TBI patients, such as caspase-3 (156, 157), soluble Fas (156, 158), cytochrome c (159–161), Bcl-2 (162), alpha II-spectrin breakdown products (163), neurofilament cleavage products (164), and HMGB1 (159, 165) offer the possibility to probe cell death pathways in human TBI patients. A particular biomarker may identify a patient population that will be most susceptible to a particular neuroprotective strategy (166). Most of the cell death biomarker studies cited above have been in CSF, with the exception of Ref. (164), which was in brain microdialysates. The latter technique provides better scope for continuous monitoring in TBI patients, particularly as CSF is not always drained, and several of the cell death biomarkers are under 100 kDa making them potentially suitable for microdialysis. Better understanding of cell death biomarkers may suggest ways to better manage patients and provide appropriate neuroprotective strategies at the most effective times.

## Biomarkers of Cerebral Circulation in SAH

Advancement of understanding of the pathophysiology of SAH has led to a considerable reduction in mortality (167). Factors that exert the strongest effects on outcomes have been identified as: age, neurological grade on admission, amount of SAH on admission CT, presence of intracerebral or intraventricular hemorrhage, aneurysm size and location, and presence of delayed cerebral ischemia (DCI) (168, 169). These prognosticators have remained unchanged since the 1990s. Nevertheless, new biomarkers of brain injury are being investigated.

Aneurysm rupture leads to intracranial hypertension and transient cerebral circulatory arrest (170, 171). Clinically, this manifests with depressed consciousness and is categorized using grading scales – currently the most important determinant of outcome. However, cerebral ischemia is known to cause activation of several key pathophysiological pathways, which may propagate primary injury, as well as increase tissue vulnerability to secondary insults, including DCI.

In excess of 40 different clinical scales have been described. The most frequently used are the Hunt and Hess scale (172) and the World Federation of Neurosurgical Societies scale (173), which describe the level of consciousness and the presence of neurological deficits (168, 169).

Imaging characteristics have always been an important tool evaluating SAH. The amount of SAH on the initial CT scan is known to be associated with development of DCI (however, less so with overall outcome) (174). The first systematic scale grading subarachnoid blood load was described by Fisher et al. (174) and remains to be the most widely used scale to date. Subsequently, numerous different classifications have been introduced with the aim of refining the predictive power, although few of these have added real clinical benefit and have not been as widely implemented (175–177).

Besides blood load, other early imaging characteristics have also been used for outcome prediction. Early CT perfusion has gained interest as a predictor of DCI risk. However, while current evidence supports CT perfusion for detecting DCI, the available data does not uniformly demonstrate that early imaging has prognostic value (178). Contrary to CT perfusion, early ischemia on MRI was shown to be a poor prognostic factor (103). However, these findings are hampered by small numbers, as well as by applicability only to poor-grade patients. Another acute imaging characteristic is global cerebral edema, present in up to 8% of patients on the initial CT, and further 12% on subsequent imaging (179). Patients with global edema have a higher incidence of cerebral metabolic crisis in the initial 12 h (180), and have less favorable outcomes (179).

**Figure 5:**
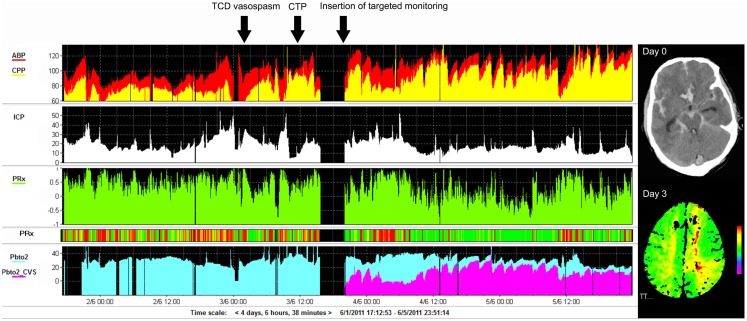
**Example of monitoring and imaging data from a patient with SAH (WFNS 5) with an AComA aneurysm, which was clipped on day 2 post-ictus**. Invasive monitoring probes including ICP and PbtO_2_, as well as an EVD, were inserted on the right side on admission. During the course of treatment, the patient developed cerebral vasospasm, which was initially detected using TCD monitoring on day 3 post-ictus (black arrow). Subsequent CTP on day 4 demonstrated a perfusion deficit in the left ACA territory (second black arrow signifies the time CTP performed), i.e., contralateral to the PbtO_2_ probe (importantly, the PbtO_2_ values were within normal range at all times, i.e., >25 mmHg and further responded to hypertension, rising to >30–35 mmHg). A further PbtO_2_ probe, targeted to the area of perfusion deficit seen on CTP, was inserted (third black arrow indicates time of insertion), which demonstrated lower/ischemic (<15 mmHg) values of PbtO_2_ (purple color on graph). ACA, anterior cerebral artery; AComA, anterior communicating artery; CTP, CT perfusion; EVD, external ventricular drain; PbtO_2_, brain tissue oxygen; SAH, subarachnoid hemorrhage; TCD, transcranial Doppler; WFNS, world federation of neurosurgical societies scale.

Serial clinical examination is not possible in poor-grade patients, hence the interest in invasive and non-invasive brain monitoring techniques, including measurements of cerebral perfusion and ICP, venous oximetry (jugular bulb catheters), brain tissue oxygen tension (PbtO_2_), microdialysis, continuous electroencephalography (EEG), and TCD. Results are best interpreted when assessed using multimodality brain monitoring protocols (Figure 5). TCD is the most common neuromonitoring modality used in SAH. Flow velocity and Lindegaard ratio are used to detect vasospasm (181, 182). Although sensitive, only 50% of patients with positive TCD have clinical symptoms. Most PbtO_2_ and microdialysis data have been extrapolated from head injury. Despite the lack of large scale studies, metabolic derangements are frequently demonstrated in patients with SAH, in particular those with cerebral edema (180). Furthermore, decreased glucose, increased LPR as well as increased glutamate levels have been associated with DCI. Microdialysis is able to predict DCI with 82% sensitivity and 89% specificity (68). Similarly, frequent episodes of cerebral hypoxia (PbtO2 < 15 mmHg) have been shown to be associated with fatal outcome (183). Importantly, metabolic derangement and hypoxia are frequently not related to intracranial hypertension nor reduced cerebral perfusion, supporting the need for multimodal monitoring as well as routine imaging (184). Other experimental methods include electrocorticography to detect cortical spreading depolarizations (SD), which when clustered can lead to an inverse hemodynamic response, and thus hypoperfusion and hypoxia, and tend to be related to DCI (185, 186). Despite these promising findings and the wealth of pathophysiological information gained, the use of monitoring in guiding management remains to be determined. In particular, the focal nature of DCI poses a challenge for all focal monitoring modalities (Figure 5) (187).

Different modalities, such as TCD, PbtO_2_, and near-infrared spectroscopy, have been used to assess the state of cerebral blood flow autoregulation–a possible homeostatic mechanism, which is thought to be affected following acute insult to the brain. The theory of the importance of autoregulation is related to the presumed dual control mechanism of cerebral blood flow, whereby, in physiological conditions, a single insult such as reduced cerebral perfusion or vessel narrowing will not produce significant alterations of blood flow due to autoregulatory compensation (188). Loss of autoregulation in the first 5 days has been found to be predictive of both unfavorable outcome as well as DCI (189, 190).

A number of biochemical compounds have been investigated as potential markers of brain injury and; however, none have been incorporated into clinical routine. Interestingly, investigated compounds can be divided into those which are thought to signify on-going brain damage/ischemia and those that are thought to play a role in the pathogenesis of DCI. Hyponatremia has been associated with cerebral infarction (191). The exact mechanism remains unknown; nevertheless, some clinicians use it as a marker of DCI. S-100beta is another compound that is known to indicate brain injury, which increases in patients after SAH (192). A recent study, looking at a panel of four markers of brain ischemia (including S-100beta), sampled within 50 h of ictus showed good predictive power for poor outcome (193). Others have studied compounds, which are thought to play a role in the pathogenesis of DCI. Notably, Endothelin-1 (ET-1), a potent vasoconstrictor, has been shown to be a marker of DCI (194). However, administration of ET-1 receptor antagonists does not improve outcome (195). Inflammation is thought to play an important role in brain ischemia after SAH, suggesting pro-inflammatory cytokines could be used as biomarkers as well as therapeutic targets. It has been shown that early increases if IL-6, both in serum and CSF, are predictive of DCI (196, 197). However, it must be noted that these findings may be contaminated by the presence of sepsis, which in itself is a poor prognosticator.

Numerous methods of stratifying brain damage after SAH have been investigated, ranging from clinical grading scales, through imaging findings and invasive neuromonitoring modalities and finally to assessment of molecular biomarkers form serum and CSF. However, despite the wealth of pathophysiological insight, few methods have gained widespread clinical acceptance. Clinical assessment with the use of grading scales remains the gold standard for outcome prognostication and decision-making. Similarly, DCI is diagnosed on the basis of neurological deterioration. TCD, angiography as well as perfusion imaging remain useful as confirmatory tools, while neuromonitoring, although showing promise is limited to only a subset of patients and is hampered by the focal nature of available probes. Further research is needed before reliable biochemical markers are available for clinical use.

## Integration of Biomarkers from Multiple Modalities with Different Timescales

Important considerations in brain monitoring are those of timescales and sampling frequency. Data from modalities such as ICP, CPP, brain tissue oxygenation, pressure reactivity, etc., are captured at high rates–e.g., from 100 Hz to once per 10 s. The Nyquist theorem (198) states that any time-variable modality should be sampled at least two times more frequently than the maximal rate of its expected changes. These sampling frequencies are thus without doubt sufficiently fast to express any underlying pathological changes whatever their timescale. In contrast, biochemical changes are, of necessity, sampled much more slowly, e.g., brain microdialysis routinely has hourly vial changes and readings, while samplings of blood and CSF for analysis are typically twice a day. Furthermore, scans such as MRI and PET are “snapshots” done usually just once or twice over the course of the patient‘s neurocritical care. The question thus arises of how best to integrate data modalities with such diverse time-bases. ICP and other rapid-sampling data can be averaged, e.g., over 60-min periods, to put them on an equal footing with the hourly microdialysis data for statistical evaluation (8, 9). There is evidence to suggest that TBI patients‘ brain chemistry in many cases evolves relatively slowly; Nelson et al. found that cerebral microdialysis data are often highly auto-correlated even up to a future 30 h, but with weak correlation to ICP and CPP (75) (also see earlier section entitled Extracellular Biomarkers of Brain Chemistry). Thus, the dominant processes monitored by microdialysis may be long-term, possibly spanning days or longer, and arguably may be better related to scans than to rapid changes evident from ICP and CPP, etc. A notable exception regarding the speed of changes in brain chemistry may be in the situation of SD where pre-clinical studies indicate rapid changes in chemistry following propagation of the SD wave (199) or in the case of seizures (200). The development of rapid/continuous online detectors for microdialysis (199), as well as for blood and CSF, may assist in getting a truer picture of temporal changes in brain chemistry.

## Conclusion and Future Prospects

Here, we have reviewed many of the different types of biomarkers that are available, or potentially available, for monitoring the human brain after severe acute traumatic and non-TBI. While only a small number of these biomarkers are in routine use, and then only in relatively specialized neurocritical care centers, there is a much scope for more of the non-routine biomarkers to play a role in clinical research studies to provide surrogate endpoints in Phase II studies. The latter are typically in small numbers of patients and are not statistically powered for clinical outcomes. In such context, intelligent selection of biomarkers can provide useful pointers toward efficacy at the biochemical level, which may assist decision-making as to which agents and/or procedures are worth pursuing into larger, much more costly and time-consuming Phase III trials. Examples of biomarker methodology are summarized in Table 1. Whether we need more biomarkers is an open question, which may be partly answered by increased use of screening techniques such as metabolomics and proteomics, which may help identify hitherto unrecognized candidate biomarkers.

Better use of the biomarkers we have identified already is certainly needed. Future advances in sensor technology such as lab-on-a-chip and replacement of current invasive brain probes with smaller, less invasive devices may help promote the monitoring of biomarkers at the bedside. Even with existing technology, part of the answer may be in advances in data handling methods, including multivariate analysis and machine learning technologies, particularly those that address temporal patterns. An essential foundation is the large databases that are continuing to accrue in specialist centers and multi-center collaborations. Currently, interpretations hinge on consideration of each of the parameters in relation to “consensus” thresholds based on collective experience among clinicians, and such thresholds are often debated. Furthermore, temporal patterns are usually evaluated visually. We are reaching the stage when simply “eyeballing” complex multimodal datasets may be insufficient to assess the true situation and prospect for the individual patient. More objective evaluation methods may enable better integration of time-variant, multi-parameter data and piece together the jigsaw puzzle of complex evidence to create an informative picture.

## Conflict of Interest Statement

Peter J. Hutchinson is a Director of Technicam. Marek Czosnyka has a financial interest in a fraction of the licensing fee for ICM+ Software (http://www.neurosurg.cam.ac.uk/pages/ICM/index.php) licensed by Cambridge Enterprise, Cambridge, UK. The other authors have no conflicts of interest concerning this review.

## Abbreviations

ABP, arterial blood pressure; ADC, apparent diffusion coefficient; ARDS, acute respiratory distress syndrome; ATP, adenosine triphosphate; BTF, Brain Trauma Foundation; CMR_glc_, cerebral metabolic rate of glucose; CMRO_2_, cerebral metabolic rate of oxygen; CNS, central nervous system; CPP, cerebral perfusion pressure; CSF, cerebrospinal fluid; CT, computed tomography; DCI, delayed cerebral ischemia; DIND, delayed ischemic neurological deficits; DTI, diffusion tensor imaging; DWI, diffusion weighted imaging; EEG, electroencephalography; ET, endothelin; FA, fractional anisotropy; FDG, ^18^F-fluorodeoxyglucose; FLAIR, fluid-attenuated inversion recovery; GABA, gamma-aminobutyric acid; GCS, Glasgow coma scale score; GFAP, glial fibrillary acidic protein; GOS, Glasgow outcome scale score; GE, gradient echo; GRE, gradient recalled echo; HPLC, high performance liquid chromatography; ICH, intracranial hemorrhage; ICP, intracranial pressure; IL, interleukin; i.v., intravenous; LDH, lactate dehydrogenase; LPR, lactate/pyruvate ratio; MMP, matrix metalloprotease; MRI, magnetic resonance imaging; MRS, magnetic resonance spectroscopy; NAA, *N*-acetylaspartate; NAAG, *N*-acetylaspartylglutamate; NAD^+^, nicotine adenine dinucleotide (oxidized form); NADH, nicotine adenine dinucleotide (reduced form); NMR, nuclear magnetic resonance; NSE, neuron-specific enolase; PbtO_2_, brain tissue oxygen tension; PCR, phosphocreatine; PET, positron emission tomography; Pi, inorganic phosphate; POCE, proton-observe carbon edited; PPP, pentose phosphate pathway; PRx, pressure reactivity index; ROI, region(s) of interest; SAH, subarachnoid hemorrhage; SD, spreading depolarizations; SWI, susceptibility-weighted imaging; TAI, traumatic axonal injury; TBI, traumatic brain injury; TCA cycle, tricarboxylic acid cycle; TCD, transcranial Doppler; TNF, tumor necrosis factor.
